# Metal Electrode Polarization in Triboelectric Nanogenerator Probed by Surface Charge Neutralization

**DOI:** 10.1186/s11671-022-03682-8

**Published:** 2022-04-03

**Authors:** Jiwon Jeong, Byungsoo Yoo, Eunji Jang, Inje Choi, Jongjin Lee

**Affiliations:** Department of Physics and Research Institute of Natural Science, Gyeongsang National University, Jinju, 52828 South Korea

**Keywords:** Surface charge, Polarization, Charge balance, Metal electrode, Triboelectric nanogenerator

## Abstract

Triboelectric nanogenerator (TENG) uses charge transfer between two asymmetric charge affinity materials such as metal and dielectrics. Metal electrode acts as charge collector from dielectrics and acts as charge transfer path to an external load, which model deals with only a net charge of metal electrode concerning electrical output. In this work, we found that metal electrode in triboelectric generator has non-negligible surface charge polarization causing open-circuit voltage difference in the model TENG system. The output voltage depends on the initial preparation conditions of the TENG for I–V measurements, even for the same measured charge densities. The measured output voltage difference with the same charge density implies that electric charges of TENG are composed of movable charges that affect current and voltage output and the bounded fixed charges that only affect open-circuit voltage.

**Supplementary Information:**

The online version contains supplementary material available at 10.1186/s11671-022-03682-8.

## Introduction

Recently, with the advance of digital technology, electronic components became less power-demanding. However, wearable devices and wireless sensor networks need inconvenient charging processes and limited operation time. Sustainable in situ power generation [1–3] or wireless power transmission systems [4, 5] are actively studied to circumvent these problems. Triboelectric nanogenerator (TENG) is considered a promising candidate for sustainable energy harvesting because of easy fabrication and plenty of operating modes [6].

TENG comprises two structural components of a triboelectric dielectric layer and metal electrodes for charge movement [6]. The efficiency of TENG is known to be governed by surface charge density of the dielectric layers [7–10], which can be enhanced by various methods of surface treatment [11], structural optimization [12], and direct prior charge injection [13, 14]. However, it is also known that obtainable surface charge is limited by dielectric breakdown through the environmental air following Paschen’s law [13, 15, 16], which was proved by enhanced charge density in vacuum operation [17]. Many works have been done to overcome this breakdown problem [18–20]. Nowadays, new approaches such as the adoption of pumping generators for the continuous high dielectric charge, charge relocation and doubling during operation appear to overcome this dielectric breakdown problem [21–23]. The Wimshurst machine is a classical triboelectric charge generator operated by relocating induced charges through a metallic neutralization bar, resulting in high net charge collection efficiency without triboelectric contact motion [24–26].

In this work, we study the surface polarization in metal electrodes which is in contact with charged dielectrics in contact-separate mode TENG (CS-TENG). When a metal plate is located in an electric field, the upper and lower surfaces have opposite polarity but are neutral in total charges. Subsequently, when one side of the charge is neutralized by any method, the plate will have a true net charge. This procedure is the basis of Wimshurst machine operation. We examined these phenomena and related them with CS-TENG measurement procedures. Finally, we will show these phenomena affect the output of our model CS-TENG system, especially open-circuit voltage.

## Methods/Experimental

### Fabrication of TENG

**Fig. 1:**
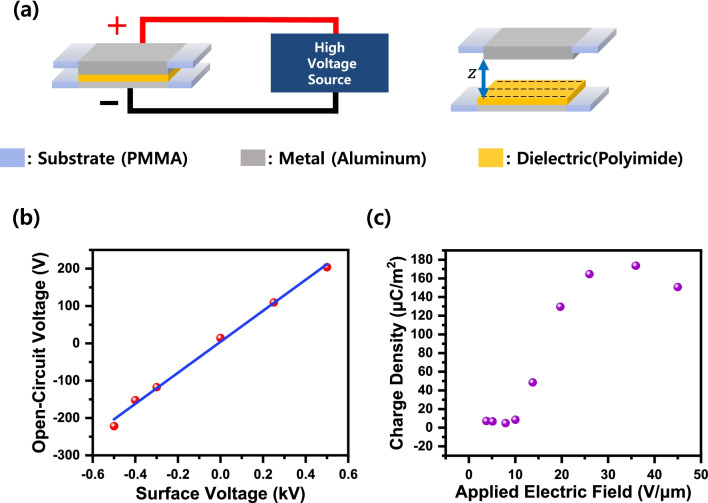
**a** CS-TENG structure and dielectric surface charge injection method for the prior charge injection. **b** Output voltage depending on the surface charge density. **c** The relationship between the applied charge injecting electric field and the charge density

The TENG used in the experiment made a contact area by attaching a polymethyl methacrylate (PMMA) substrate ($2 \times 2\, {\text{cm}}^{2}$) to a PMMA substrate ($4 \times 4\, {\text{cm}}^{2}$). As an electrode, the 50-μm-thick aluminum (Al) tape is attached to the PMMA substrate ($2 \times 2 \,{\text{cm}}^{2}$) and the 50-μm-thick polyimide (PI) tape is stuck to the Al tape electrode as the dielectric layer. A typical model of CS-TENG is depicted in Fig. 1a. Four springs fixed to the edges are used to maintain the distance between the bottom substrate, which consists of the dielectric and the electrodes, and the top substrate, which consists of the electrodes.

### Characterization and Measurement

The output characteristics of the TENG are controlled by a prior charge injection method using an electric field generated from a high-voltage power supply. In the structure of CS-TENG, when a high direct current (DC) electric field is applied on both electrodes, dielectric breakdown occurs in the air layer between the electrode and the dielectric. Subsequently, the ionized charge will be injected onto the dielectric layer. The injected surface charge can be maintained for a period [13, 14]. As shown in Fig. 1b, the surface and output voltage show a linear relationship in the electric field and determine the output adjusting the surface voltage. As the strength of the electric field increases, the charge density of the dielectric surface also increases. However, if the charge density rises above a certain level, the charge density decreases after that. This phenomenon shows that the charge density is limited by the dielectric breakdown occurring on the surface (Fig. 1c). CS-TENG within the section where a constant charge density is maintained, and the charge density of the electrode surface neutralization process performs while controlling. The operation of CS-TENG carries out through the contact-separation process using a solenoid (Shindengen, M080117SS). A force sensor (Marveldex, RA18) attaches to the lower part of the CS-TENG to monitor whether a uniform force was applied. The electrical output characteristics were measured using an electrometer (Keithley, 6514), the open-circuit voltage ($V_{{{\text{OC}}}}$) and short-circuit charge ($Q_{{{\text{SC}}}}$). The surface voltage is measured using a non-contact electrostatic field meter (SIMCO, FMX-004), and the analog output is connected to an oscilloscope (Keysight, DSO-2014A) for data acquisition.

## Results and Discussion

### Open-Circuit Voltages Depending on Initial Charge Balancing

**Fig. 2:**
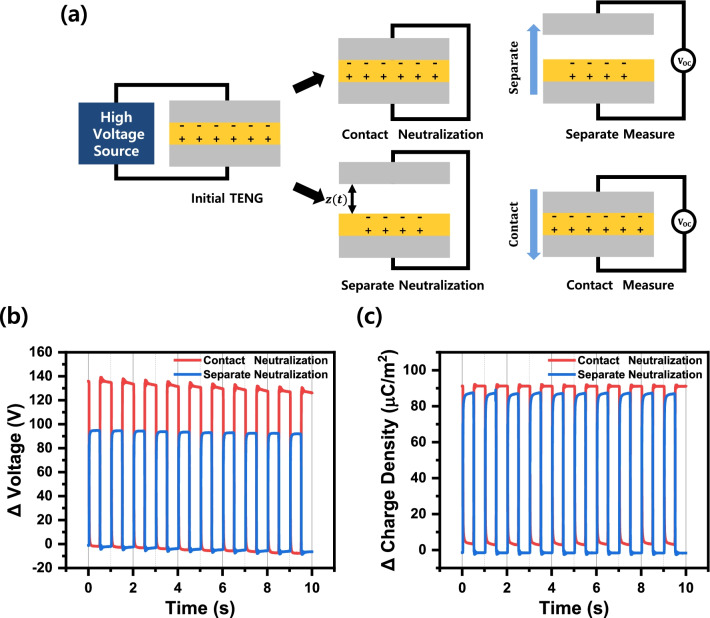
**a** Two different electrode charge balancing for TENG output measurement, **b** output voltage, note the peak and bottom sequence depending on the charge balancing method, and **c** the charge density. For easy comparison, we take absolute values for the voltage and charge density

Experiments were performed to investigate the effect of metal electrodes on the output in TENG. To examine only the effect of the metal electrode, we controlled the dielectric charge density using the prior charge injection method. Then, we neutralized different polarity charges by making both metal electrodes an equipotential surface. As shown in Fig. 2a, both electrodes were short-circuited at contact and separate state, and we measured I–V afterward. The charge balancing process of connecting the upper and lower electrodes makes both surfaces equipotential by the charge transfer, redistributing the surface-bound charges. This process is the same as setting the reference voltage in measurement, setting the potential between the electrodes to be 0 V. Figure 2b, c shows the change in output voltage and charge density change due to the difference in the initial charge balancing method. After initial contact mode balancing to the reference voltage (Δ*V* = 0), the voltage change is a negative voltage of 92.46 V. And initializing at the separate mode charge balance to the reference voltage (Δ*V* = 0), the voltage change is a positive voltage of 133.92 V. Separate-mode charge balancing shows a difference in the output voltage of about 45% larger than that of the contact method. These voltage changes are reversible depending on the order of the charge balancing method, while the charge densities were the same. Specifically, the charge density measured by the short-circuit current method is 87.56 μC/m^2^ as negative polarity in contact mode and 85.49 μC/m^2^ as positive polarity in separate mode electrode charge balancing, resulting in less than 5% difference. Two different neutralization methods showed almost identical charge densities.

Since the moving distance of the contact-separation process is identical, the capacitance change during TENG operation is also the same. Consequently, the same net charge and exact capacitance change during one period of contact separation should render similar voltage following *V* = *CQ* relation. But different open-circuit voltage implies non-negligible additional bound charge should exist and makes voltage difference depending on the initial state of the electrode.

### Charge Polarization in the Metal Electrode

The electric field generated by the polarization in the dielectric layers of CS-TENG induces electric charges on the metal electrodes, making a potential difference between the top electrode (TE) and bottom electrode (BE) as measured in apparent voltage output. In addition to the inter-electrode potential difference, surface polarization can also be induced inside each metal electrode.

**Fig. 3:**
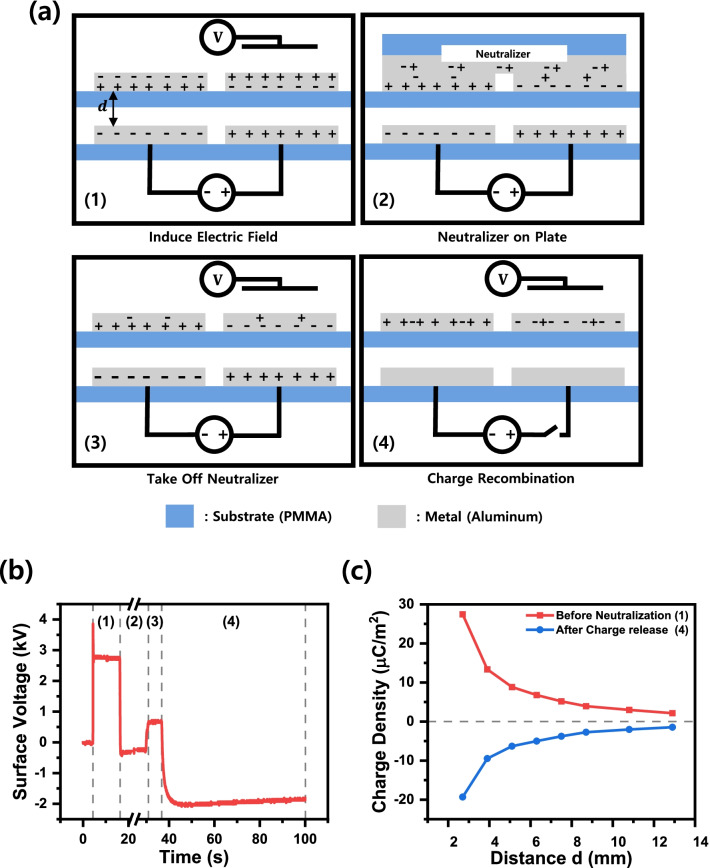
Polarization model experiment of the metal electrode in the electric field. **a** The process of net charge in metal polarization and **b** metal’s surface voltage. **c** The charge density of the metal generated by the electric field [1] and the net charge is generated [4] according to the distance from the plate electrode

To understand the metal surface charges induced by the external electric field, we conducted a model experiment. The model experiment is shown in Fig. 3a. Attach Al tapes as electrodes side by side with a 30 mm distance on a PMMA substrate. An additional layer of identical electrodes on the insulating substrate was positioned on top of the first layer with regular heights. The charge redistribution experiment was done as follows: First, connect a high voltage source to the bottom plate electrodes, thus applying an electric field in nearby space. The top electrode’s surface voltage generated by polarization induced by the electric field was measured using an electrostatic field meter. Second, a metal plate was used to shorten the two top electrodes. The metal plate neutralized different charges induced by electric fields on top of the electrodes because the two surfaces become equipotential during this process. Third, after separating the metal neutralizer, we measure the surface voltage of the top electrodes again. Finally, turn off the high-voltage source to remove the electric field and to release captured charges. We measured the surface voltage after charges were redistributed. During the process, the surface voltage changes, as shown in Fig. 3b. Because the top electrode is isolated from the bottom electrode and the applied voltage is not so high to induce air discharge (~ 0.4 kV/mm which is less than discharge limit of 3 kV/mm [27]), there is no net charge transfer. Therefore, the measured voltage was caused by the top metal surface polarization. Because the Al electrode is thick enough to screen the lower surface charge, measured voltages came from the upper surface since we measured the upper surface in non-contact mode. The measured potential is null at the second step because we cannot access the surface voltage because of the top-covered metal neutralizer. Then, a sudden decrease of surface voltage appears in the third step because top bound charges recombined and small remaining amounts exist. Finally, when the high-voltage source connected to the BE turns off, the electric charges fixed at the lower surface of the TE are released from the electric field. Considering the first polarized surface voltage and remaining reduced surface voltage just after neutralization, we can find that polarity reversed surface voltage is the difference between the previous two values, which confirms our explanation. In this process, the residual charge on the upper surface and the fixed charge on the lower surface are redistributed to the metal net charge. Figure 3c shows the charge density measured before and after the neutralization process by varying the distance between the upper and lower plates. We verified that both charge densities decrease inverse proportionally with increasing inter-plate distance. Through the sequential procedure, we confirmed top and bottom of each electrode bears different charges induced by nearby field sources, which are bound to each other but can be released/removed along with equipotential lines. At the strongly bounded side, remaining net charges are captured and finally released when the external field sources are removed. Our model experiment explains that the electrode's surface charge generated by the metal polarization and additional shortening (neutralization) of the circuit can be the source of potential difference.

### Polarization of Metal Electrode in CS-TENG

The previous experiment showed that polarized charges exist on both sides surfaces of the metal electrode. The neutralization between two different polarities of just one surface makes remaining net charges. When we try to measure the output voltage of our CS-TENG, we need to set the initial reference point of the voltage measurements. Technically, we can achieve this in two different ways. First, we set the reference point as any arbitrary point before the measurement and measure the voltage difference between two points from that arbitrary reference point. This reference set does not need any charge movement but requires a very high input range to cover the reference point and measurement objects. Second, the measurement unit adjusts internal potential along with the outer object in an initial stage of voltage measurement. This can be done by compensating the internal voltage source to match an external object with an equal level. Internal charge source provided balancing charges, and two terminals became electrically equipotential. Most high-voltage electrometer adopts the second method, specifically also our measurement tool. For this reason, at the beginning of voltage measurements, the procedure that we set the reference point as 0 V is the same as we neutralize the two terminal's potential difference to the equipotential surface value of 0 V.

**Fig. 4:**
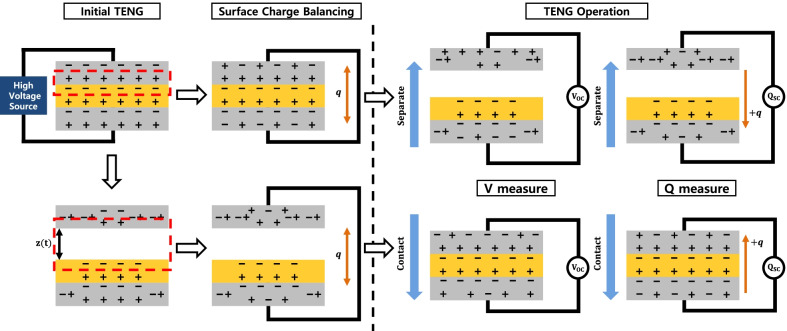
Schematic diagram of electric charges movement between the metal electrodes under the different charge balancing conditions in the same TENG structure

As we examined our neutralization model, some surface charge should remain in our model CS-TENG operation. Thus, the bound surface charge distribution in metal electrodes should be considered during the TENG operation. We suggest a surface charge polarization model in which the open-circuit voltage changes according to the charge balance conditions of the CS-TENG. In CS-TENG composed of TE, dielectric, and BE, the electric field is reduced when *z*(*t*) increases in this referencing stage. The weakened electric field reduces the metal polarization. The polarization of TE separated from the dielectric by *z*(*t*) becomes smaller than BE. We depicted the reduced polarization as shown in Fig. 4. In this stage, the electrode neutralization process occurs on both electrodes for surface charge balancing. When the top of TE and bottom of BE are connected, the surface charge of the two electrodes moves for electrical balance. The amount of moving charge will be changed according to the surface charge, whereas the polarization is influenced by *z*(*t*). When we measure the open-circuit voltage during the TENG operation, the charge transfer between the two electrodes is blocked due to the instrument’s high impedance. Thus, the remained surface charge that is un-balanced during the initial stage is fixed to the electrode. In this state, the voltage output follows capacitance changes during the contact-separate operation of CS-TENG. On the contrary, when we measure the short-circuit current, charges are free to move between the two electrodes. Regardless of the initial charge balancing conditions, the amount of the movable charges will be the same because the balancing condition only affects bound surface charges. During the contact and separate operation of the TENG, we observed that moving charges/charge densities are not affected by initial balancing. Still, open-circuit voltages depend on the initial balancing conditions.

**Fig. 5:**
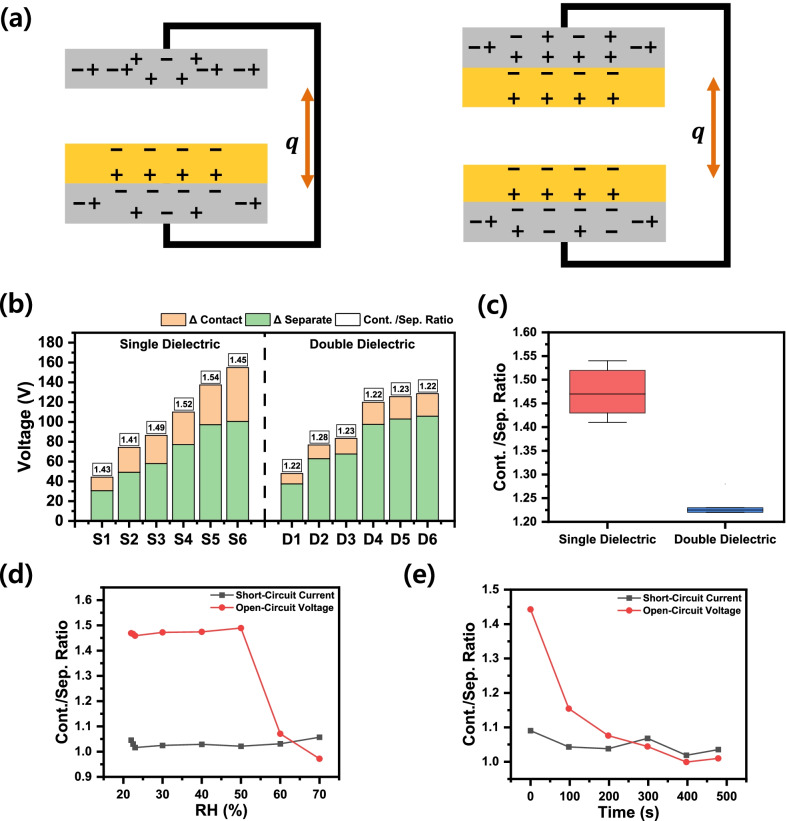
**a** Schematic diagrams of two different charge models of single TENG and double TENG structures. **b** Output voltage varies according to charge balancing conditions, but the ratio between the two balancing conditions is almost the same regardless of the varying output voltage. **c** Summary of the ratios in **b**. Contact/separate ratio of open-circuit voltage and short-circuit current under **d** different humidity conditions and **e** with extended operation time

To verify the fixed charge contribution to the open-circuit voltage, we prepared two different types TENGs. One is called a single-dielectric TENG (‘single TENG’) composed of metal-to-dielectric and metal layers. Another is called a double-dielectric TENG (‘double TENG’) composed of metal-to-dielectric and dielectric-to-metal layers, as shown in Fig. 5a. Even though the output voltage varies according to the initial conditions, the tendency of a decrease in the separate-balancing method consistently appears. When comparing contact and separate balancing voltages ratios, the voltage of ‘single TENG’ changed by about 41 to 54%, but in the case of ‘double TENG’, it changed by 22 to 28%. The constant ratio on various output voltages depending on TENG device types means that the proportion of fixed and movable charge is governed by not the number of movable charges, but the geometry of the devices (Fig. 5b). In the ‘single TENG’ device, the polarization of the TE without dielectric layer is greatly affected by the distance to the charge-bearing dielectric layer in the separate state compared to the BE.

In contrast, in the ‘double TENG’, both electrodes are in contact with the dielectric layers. Consequently, metal electrodes in ‘double TENG’ are adjacent to charged dielectrics and highly affected by the adjacent electric field, not by the varying the far-field of the counter dielectric layer. Thus, surface charges in both electrodes are evenly fixed and less affected by different initial charge balancing. As shown in Fig. 5c, average contact/separate charge balancing open-circuit voltage ratios bear two different ranges. To figure out the characteristics of these fixed charges, we measured the ratios of the ‘single TENG’ device with different operating conditions. Figure 5d shows the contact/separate ratios with varying humidity conditions. In low-humidity conditions, the ratio has constant values without significant changes. In high-humidity conditions, the contact/separate ratio of open-circuit voltage rapidly decreases. Similar behaviors can be seen in the extended operating times of Fig. 5e. These results also confirm the existence of the fixed charges. Though their binding is enough to survive during the initial charge balancing process, they experience atmospheric discharge and show slow decaying properties during extended TENG operation (Additional file 1: Figures S1–S3).

## Conclusions

This study showed that the electric field of charged dielectric causes polarization of metal electrodes and can generate net charge through an appropriate charge neutralization process. We analyzed that charge balancing conditions can change the voltage output during the operation of CS-TENG, which is due to the different surface polarization of the metal electrode even with the exact movable charges and capacitance.

This potential difference originated from the fixed charges, which remained as a result of the metal electrode polarization. As the distance increases during the separate operation of TENG, the electric field acting on the metal electrode weakens and reduces the polarization. This effect is dominant for the single TENG device having a dielectric layer attached to only one metal electrode. With the symmetrical structure of the double TENG, both electrodes have fixed charges captured by adjacent dielectric layers and are equally less affected by the inter-electrode electric field changes. Rather than one electrode being attached to a dielectric layer, having dielectric layers on both sides increases the fixed charges, which works profitably for the output of TENG.

## Supplementary Information

**Additional file 1: Fig. S1.** (a) Current output according to movable charge in TENG and (b) Δ current output according to Δ movable charge in TENG depending on contact charge balancing process (CBP) and separate charge balancing process (SBP). **Fig. S2.** (a) voltage output according to movable charge and (b) Δ voltage output according to Δ movable charge in CS-TENG depending on CBP and SBP. **Fig. S3.** Contact/separate ratio of open-circuit voltage and short-circuit current under conditions different surface voltage when air breakdown occurred in high surface voltage.

## Data Availability

The datasets used and/or analyzed during the current study are available from the corresponding author on reasonable request.

## Abbreviations

TENG: Triboelectric nanogenerators
CS-TENG: Contact-separate mode TENG
PMMA: Polymethyl methacrylate
Al: Aluminum
PI: Polyimide
TE: Top electrode
BE: Bottom electrode
single TENG: Single-dielectric TENG
double TENG: Double-dielectric TENG
