# Supplementary material for: Metal Electrode Polarization in Triboelectric Nanogenerator Probed by Surface Charge Neutralization
Source: Nanoscale Res Lett. 2022 Apr 3;17:42. doi: 10.1186/s11671-022-03682-8 (PMC8976868; doi:10.1186/s11671-022-03682-8)
Supplement: Supplementary file 1 — Additional file 1: Fig. S1. (a) Current output according to movable charge in TENG and (b) Δ current output according to Δ movable charge in TENG depending on contact charge balancing process (CBP) and separate charge balancing process (SBP). Fig. S2. (a) voltage output according to movable charge and (b) Δ voltage output according to Δ movable charge in CS-TENG depending on CBP and SBP. Fig. S3. Contact/separate ratio of open-circuit voltage and short-circuit current under conditions different surface voltage when air breakdown occurred in high surface voltage. [file 11671_2022_3682_MOESM1_ESM.docx]

**Supplementary Information**

**Metal electrode polarization in triboelectric nanogenerator probed by surface charge neutralization**

Jiwon Jeong, Byungsoo Yoo, Eunji Jang, Inje Choi, Jongjin Lee^*^

* Author to whom correspondence should be addressed (J. Lee)

Tel.: +82-55-772-1401

E-mail address: [bandy1@gnu.ac.kr](mailto:bandy1@gnu.ac.kr)


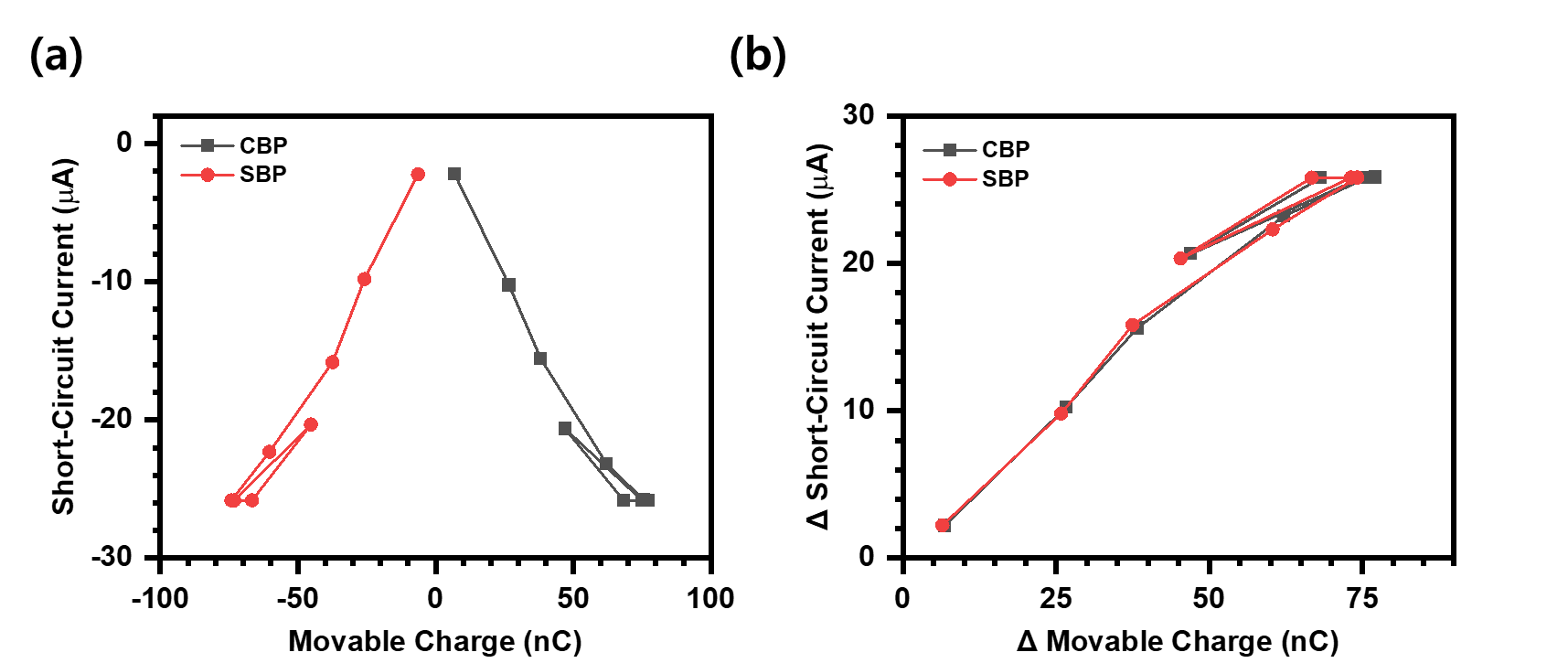


Fig. S1. (a) Current output according to movable charge in TENG and (b) Δ current output according to Δ movable charge in TENG depending on contact charge balancing process (CBP) and separate charge balancing process (SBP).


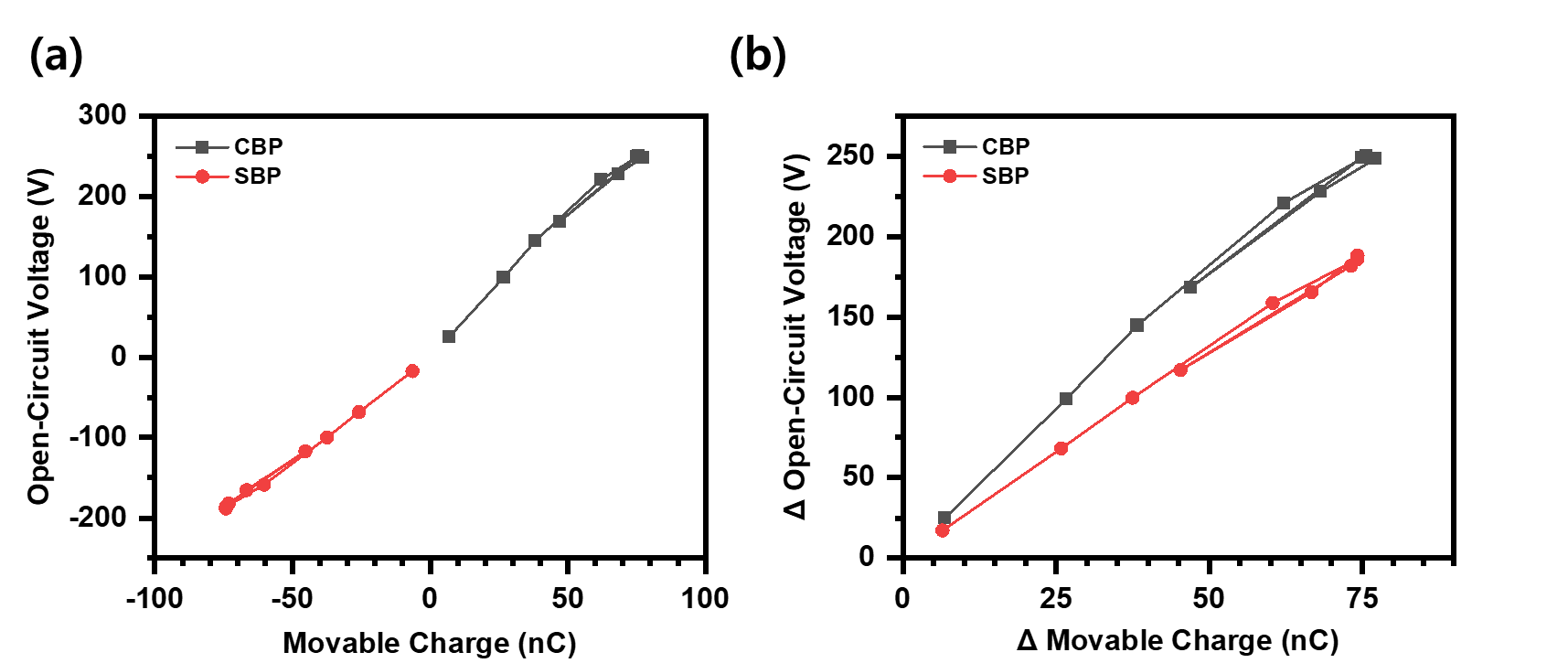


Fig. S2. (a) voltage output according to movable charge and (b) Δ voltage output according to Δ movable charge in CS-TENG depending on CBP and SBP.

Fig. 3. Contact/separate ratio of open-circuit voltage and short-circuit current under conditions different surface voltage when air breakdown occurred in high surface voltage.
